# Fatal Effects of Ether Vapour in a Case of Lithotomy

**Published:** 1847-05

**Authors:** Roger Nunn

**Affiliations:** Surgeon to the Colchester and Essex Hospital


*Fatal Effects of Ether Vapour in a case of Lithotomy.* By Roger Nunn, Esq., Surgeon to the Colchester and Essex Hospital.—At a time when the attention of both the medical profession and the public is being called to the influence of the ethereal vapour as an agent in deadening pain during surgical operations, you may probably consider the accompanying case of sufficient interest to be admitted into your columns.

On Friday, the 12th inst., I operated upon Thomas Herbert, ætat. 52, the subject of stone in the bladder, in the presence of most of the medical gentlemen of the town and neighbourhood. The ether was exhibited by my colleague, Dr. Williams, who considered the patient to be sufficiently under its influence after having inhaled it seven or eight minutes, at the end of which time I commenced the operation. There was neither difficulty nor loss of time in cutting into the bladder; but, having done so, some little delay occurred in grasping the stone, which was small, very flat, and lying in the posterior part of the bladder; the delay was also increased by the extremely relaxed state of the bladder itself which seemed to fall in folds on the forceps, and to cover the stone. The time occupied from the commencement of the operation to the period when the man was unbound, was ten minutes, during which the ether was administered at intervals. The patient was placed fully under its influence, and the breathing first became heavy, and ultimately stertorous. He recovered, however, from its effects after a short time, and continued in a quiet passive state, but without decided reaction, for 24 hours. At this period he had a chill, which lasted for nearly twenty minutes. Mr. Taylor (the house-surgeon,) immediately gave him two ounces of brandy, with an equal quantity of water, after which the patient remained in a dozing state till 8 o'clock, P. M., when the house-surgeon considered it necessary to send for me, as a state of complete prostration or collapse had ensued. I ordered small quantities of brandy and water (equal parts,) with arrow-root, at intervals, wrapped him in hot blankets, placed hot bottles in the bed, &c. This treatment was kept up till 9 o'clock the following morning, when ammonia was given alternately with the before-mentioned stimulus. The patient seemed incoherent from 8 o'clock, P. M., of Saturday till 9 A. M., of the following day, when symptoms of slight reaction appeared. At a consultation of the medical staff, which was held at the time, it was determined that the same treatment should be continued, (modified according to circumstances,) and that, in addition, a stimulating injection should be administered. (The effects of the injection were to increase slightly the frequency of the heart's pulsation, but without exciting his nervous energies.) From this time he gradually sank, and died at 5 o'clock, P. M., being sensible to the last.

I should here mention that the small vessels which are necessarily divided in making the first incision showed much inclination to bleed, owing, I imagine, to their want of contractile power. I therefore, to prevent any unnecessary hæmorrhage, secured them immediately after the patient was put to bed, so that he did not lose much blood.

*Postmortem* (67 *hours after death.*)—Membranous congestion of the brain, but no effusion; brain firm; lungs permeable throughout, anteriorly ex-sanguineous, posteriorly engorged; heart flaccid, of a natural size, and nearly empty; the left kidney pale; the right, slightly congested. The bladder and the adjoining parts presented the usual aspect after an operation.

***I would mention that the blood throughout the whole vascular system was in a perfectly fluid state.***

It is not my intention or inclination to attribute the loss of my patient wholly to the influence of the ether which was administered in this case, nor hastily to decry its use under all circumstances connected with surgical operations; but still I feel called upon to bring before the notice of my medical brethren the effects which resulted from its exhibition in this instance, that the profession may judge, from the recital of an unsuccessful case, how far it may be considered safe to employ ether generally as a means of preventing the pain otherwise inseparable from the physical lesion. The suffused eye, livid lips, and stertorous breathing, accompanied, first, by convulsive struggles, and next by a sudden cessation of all motion, are often indicative of the effects of the vapour; and these were not altogether absent in the present instance: still, I felt myself justified in employing it, from the published accounts of many successful cases, and the sanction of my colleagues and numerous friends around me. In prosecuting the operation, there was nothing peculiar to attract my attention, or to lead me to consider the patient's physical condition different from that of those on whom I had before operated, until I had reached the bladder, when I can but attribute the difficulty in seizing the stone to the apparently collapsed state of that viscus which had fallen in folds over the calculus, and so prevented its being grasped by the forceps. I must not, however, omit to mention the fact that the patient expressed no signs of suffering during the operation. Thus far, therefore, it may be said the ether fulfilled its intended office; but I think another question is involved, viz. whether the artificial means thus employed may not produce very serious depressing effects on the nervous system, depriving a patient of that reactive power so necessary to the reparative process. Has not a patient, after the administration of ether, a double shock to overcome—that produced by the vapour superadded to that of the operation itself? Does not the history of the postmortem examination bear out the suspicion of the depressing influence of this inhalation?—*positively*, from the still fluid state of the blood (although the body was not opened for 67 hours after death,) and from the flaccid state of the heart; *negatively*, from the fact that the inspection detected no indications of violence done to the parts that could explain the rapid dissolution which ensued, and that *there was no evidence of nature having made the slightest effort towards local reparation.* Pain is doubtless our great safeguard under ordinary circumstances; but for it we should be hourly falling into danger; and I am inclined to believe that pain should be considered as a healthy indication, and an *essential* concomitant with surgical operations, and that it is amply compensated by the effects it produces on the system as the natural incentive to reparative action.

I trust that the publication of this unsuccessful case may lead to the publication of many others which have occurred, so that the profession may not be led away by the erroneous supposition that the prevention of pain is so vital a desideratum in operative surgery.

*** Operators have hitherto fallen into the error of looking only to one side of the question. The profession is indebted to Mr. Nunn for placing in so strong a light the danger which may occasionally arise from the use of ether vapour. We have hitherto had a run of successful cases: it is now time that our correspondents should pause in their records of successful cases, and look to the possible danger.

*London Med. Gaz.*

